# Supplementary figures and images for: iTRAQ-based comparative proteome analyses of different growth stages revealing the regulatory role of reactive oxygen species in the fruiting body development of Ophiocordyceps sinensis
Source: PeerJ. 2021 Mar 3;9:e10940. doi: 10.7717/peerj.10940 (PMC7936569; doi:10.7717/peerj.10940)

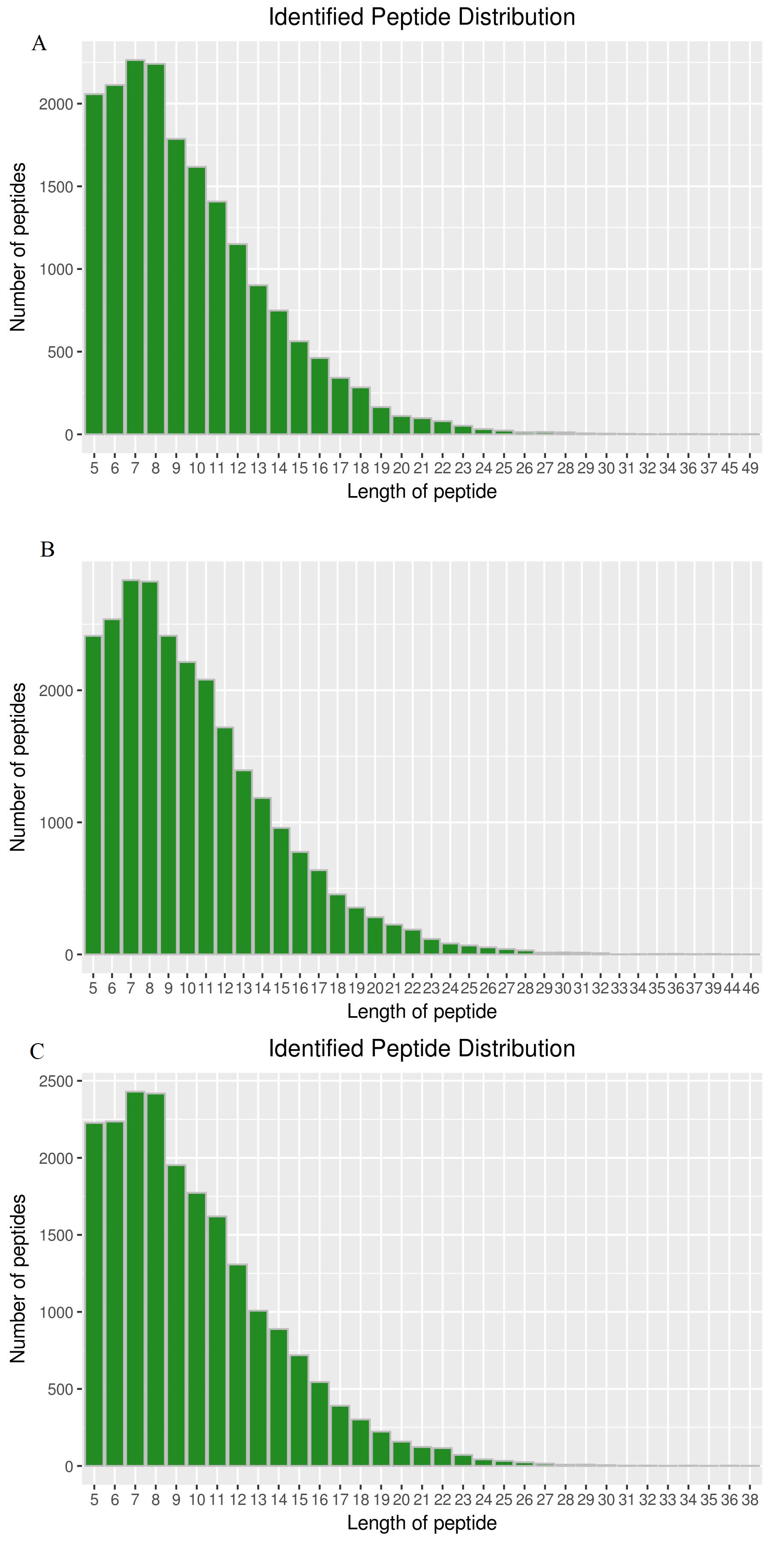

Supplement: Figure S1 [file peerj-09-10940-s001.jpg]

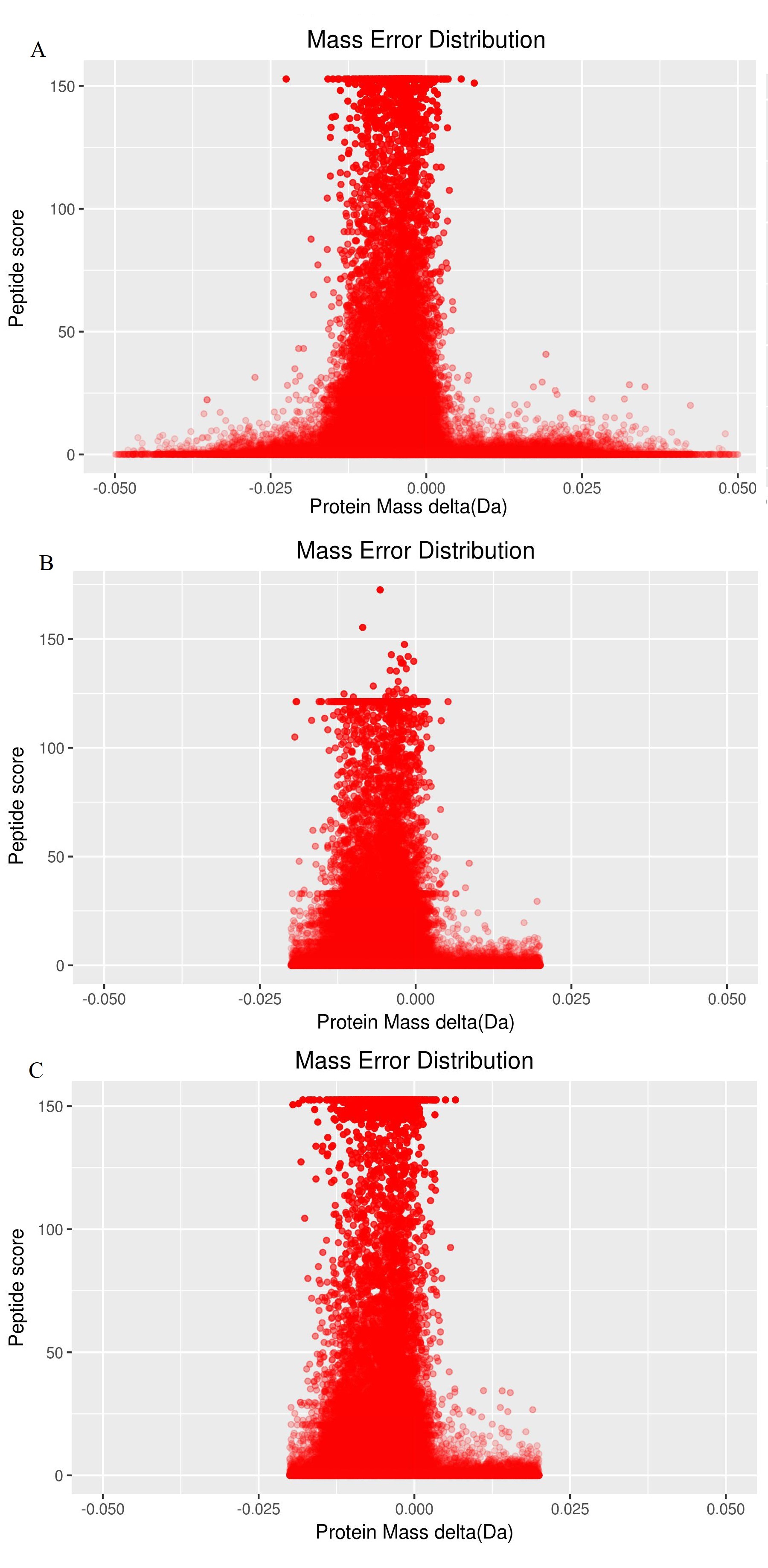

Supplement: Figure S2 [file peerj-09-10940-s002.jpg]

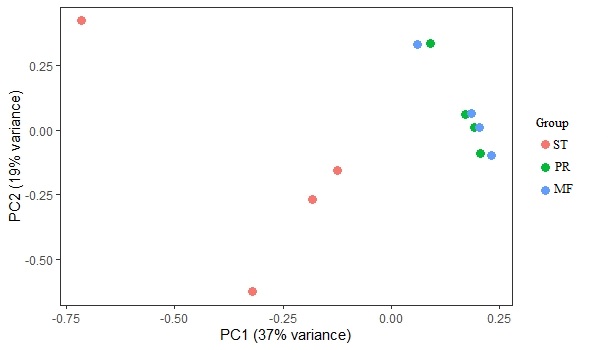

Supplement: Figure S3 [file peerj-09-10940-s003.jpeg]
