# Supplementary material for: iTRAQ-based comparative proteome analyses of different growth stages revealing the regulatory role of reactive oxygen species in the fruiting body development of Ophiocordyceps sinensis
Source: PeerJ. 2021 Mar 3;9:e10940. doi: 10.7717/peerj.10940 (PMC7936569; doi:10.7717/peerj.10940)
Supplement: Table S6 [file peerj-09-10940-s008.docx]

| **Table S6. Putatively crucial proteins in the fruit-body development.** | | | | | | | |
| --- | --- | --- | --- | --- | --- | --- | --- |
| **Proteins involved in amino acid / protein synthesis and degradation** | | | | | | | |
| **Protein ID** | **ST vs. PR** | ***p-value*** | **PR vs. MF** | ***p-value*** | **ST vs. MF** | ***p-value*** | **Annotation** |
| T5ABF2 | 1.11 | 0.021 | 0.71 | 0.0006 | 0.79 | 0.002 | phospho-2-dehydro-3-deoxyheptonate aldolase |
| T5AKZ2 | 0.51 | 0.006 | 0.81 | 0.035 | 0.41 | 0.002 | Phospho-2-dehydro-3-deoxyheptonate aldolase |
| T4ZXQ6 | 2.75 | 0.012 | 0.96 | 0.795 | 2.66 | 0.014 | Serine protease |
| T5AL30 | 1.95 | 0.007 | 1.03 | 0.761 | 1.99 | 0.005 | 26S proteasome non-ATPase regulatory subunit |
| T4ZXD0 | 2.33 | 0.021 | 1.08 | 0.508 | 2.52 | 0.015 | 26S proteasome complex ubiquitin receptor, subunit Rpn13 |
| T5AFG8 | 1.74 | 0.001 | 1.05 | 0.543 | 1.83 | 0.001 | 26S proteasome regulatory subunit RPN1 |
| T5A611 | 1.30 | 0.016 | 1.24 | 0.054 | 1.62 | 0.0008 | Proteasome subunit alpha type |
| T5AE86 | 1.68 | 0.015 | 1.07 | 0.542 | 1.79 | 0.015 | Proteasome component C5 |
| T5AFI6 | 1.54 | 0.0005 | 0.97 | 0.599 | 1.49 | 0.0007 | Proteasome endopeptidase complex OS |
| T5AA86 | 1.781 | 0.022 | 0.87 | 0.222 | 1.56 | 0.046 | Proteasome subunit  beta |
| T5A8T1 | 3.39 | 0.003 | 1.08 | 0.403 | 3.67 | 0.003 | Proteasome subunit beta |
| **Proteins involved in energy metabolism** | | | | | | | |
| T5A666 | 3.33 | 0.0006 | 1.04 | 0.659 | 3.45 | 0.0005 | Succinate--CoA ligase (ADP-forming) α subunit |
| T5A5Y2 | 1.99 | 0.0001 | 1.09 | 0.253 | 2.19 | 0.000 | Isocitrate dehydrogenase subunit |
| T4ZWD4 | 2.38 | 0.0000 | 1.11 | 0.294 | 2.63 | 0.000 | pyruvate dehydrogenase α subunit |
| **Heat shock proteins responded to environmental stress** | | | | | | | |
| **Protein ID** | **ST vs. PR** | ***p-value*** | **PR vs. MF** | ***p-value*** | **ST vs. MF** | ***p-value*** | **Annotation** |
| T5A2W7 | 0.71 | 0.002 | 1.27 | 0.005 | 0.91 | 0.341 | Hsp70 |
| T5ADA9 | 0.62 | 0.046 | 0.78 | 0.003 | 0.48 | 0.008 | Hsp70 |
| T5AGM5 | 1.80 | 0.012 | 0.51 | 0.08 | 0.92 | 0.743 | Hsp30 |
| T5AKH0 | 1.70 | 0.002 | 1.12 | 0.424 | 1.90 | 0.001 | Hsp DanJ |
| T5A0N1 | 1.18 | 0.031 | 1.26 | 0.017 | 1.50 | 0.001 | Hsp OS |
| T5AEC8 | 0.85 | 0.021 | 1.50 | 0.000 | 1.27 | 0.006 | Hsp 90 |
| **cAMP/ MAPK signal pathway in the development** | | | | | | | |
| **Protein ID** | **ST vs. PR** | ***p-value*** | **PR vs. MF** | ***p-value*** | **ST vs. MF** | ***p-value*** | **Annotation** |
| T5A9X0 | 2.13 | 0.0003 | 1.02 | 0.87 | 2.16 | 0.0006 | Gβ subunit |
| T5AF08 | 1.62 | 0.0005 | 1.17 | 0.014 | 1.90 | 0.0001 | KH domain RNA-binding protein |
| T5AKF5 | 1.60 | 0.002 | 1.27 | 0.005 | 2.03 | 0.0005 | Ras type protein |
| T5AA69 | 1.57 | 0.0003 | 1.13 | 0.068 | 1.77 | 0.0002 | Ras type protein |
| T5A7J6 | 1.43 | 0.004 | 1.02 | 0.817 | 1.46 | 0.002 | protein kinase like protein |
| T5ANJ5 | 2.00 | 0.115 | 0.88 | 0.142 | 1.77 | 0.019 | cAMP-dependent protein kinase regulatory subunit |
| T5AGS5 | 0.43 | 0.004 | 0.94 | 0.0654 | 0.41 | 0.007 | serine / threonine-protein phosphatase |
| **Peroxisome proteins** | | | | | | | |
| **Protein ID** | **ST vs. PR** | ***p-value*** | **PR vs. MF** | ***p-value*** | **ST vs. MF** | ***p-value*** | **Annotation** |
| T5A9T9 | 2.10 | 0.042 | 1.79 | 0.048 | 3.77 | 0.015 | AhpC/TSA family protein |
| T5A6F1 | 1.27 | 0.004 | 0.68 | 0.008 | 0.86 | 0.156 | Superoxide dismutase |
| T5A5N4 | 1.65 | 0.0008 | 0.62 | 0.016 | 1.02 | 0.856 | Catalase OS |
| T5AQC4 | 1.67 | 0.0008 | 0.80 | 0.000 | 1.35 | 0.008 | Long-chain acyl-CoA synthetases & Acyl-protein synthetase |
| T5AJH5 | 0.67 | 0.158 | 0.64 | 0.021 | 0.45 | 0.016 | Superoxide dismutase |
| T5AL96 | 1.87 | 0.0008 | 0.60 | 0.159 | 1.14 | 0.365 | Catalase OS |
