# Supplementary material for: iTRAQ-based comparative proteome analyses of different growth stages revealing the regulatory role of reactive oxygen species in the fruiting body development of Ophiocordyceps sinensis
Source: PeerJ. 2021 Mar 3;9:e10940. doi: 10.7717/peerj.10940 (PMC7936569; doi:10.7717/peerj.10940)
Supplement: Table S7 [file peerj-09-10940-s009.docx]

|  | **Seq ID** | **Primer F** | **Primer R** |
| --- | --- | --- | --- |
|  |  |  |  |
| 1 | T5A6F1 | 5’- GACTCTGCCACAGACGACTAC -3’ | 5’- CCAGAAGAGGGAGTGGTTGAC-3’ |
| 2 | T5AFX3 | 5’- GACGATGGGTCGCTAGATGG-3’ | 5’- GTCCGGGAACTCGATAGCTG-3’ |
| 3 | T5ALL5 | 5’- CCTTTACCCCTCCCGAACAG -3’ | 5’- AATCCTTACCCGCGGTTCTC-3’ |
| 4 | T5AKK7 | 5’- GTGTGGCAACTTACAGCGTG-3’ | 5’- GTGTAGCTAAGCGTGACCGT-3’ |
| 5 | T5A5N4 | 5’- GCGCCTACGTCACAATCAAG-3’ | 5’- TCGTAGTTCATCAGGTTCAGG-3’ |
| 6 | T5A212 | 5’- TGTCCAACCAGGCCATTACC -3’ | 5’- AGCACGAGGACGGAAAAGAG -3’ |
| 7 | 18Srna(inner control) | 5’-CTCGTAGTTGAACCTTGGGC-3’ | 5’-AACAAAATAGAACCGCGCG-3 |
